# Incidence of cystoid macular edema following routine cataract surgery using NSAIDs alone or with corticosteroids

**DOI:** 10.5935/0004-2749.20200010

**Published:** 2020

**Authors:** Keith A. Walter, Roland Y. Lee, Kevin Chen, Chris Komanski

**Affiliations:** 1 Department of Ophthalmology, Wake Forest University School of Medicine, Winston-Salem, North Carolina, USA; 2 Department of Ophthalmology, University of Missouri School of Medicine, Kansas City, Missouri, USA

**Keywords:** Phacoemulsification, Cystoid macular edema, Ketorolac, Prednisolone, Anti-inflammatory agents, non-steroidal, Facaoemulsificação, Edema macular cistóide, Cetorolaco, Prednisolona, anti-inflamatórios não esteroides

## Abstract

**Purpose:**

To evaluate the rate of cystoid macular edema development among cataract surgery patients on four different therapeutic regimens.

**Methods:**

The present study is a retrospective analysis of 5,380 eyes following uncomplicated phacoemulsification at Wake Forest University. The study period went from July 2007 to December 2012. Patients received one of four regimens, as follows: postoperative generic ketorolac 0.4% and prednisolone 1%, postoperative name-brand ketorolac 0.45% and prednisolone 1%, postoperative bromfenac 0.09% and prednisolone 1%, preoperative and postoperative bromfenac 0.09% alone. A statistical analysis was performed to assess the differences in rate of cystoid macular edema development among the four different therapeutic regimens. The diagnosis of cystoid macular edema required worsening of vision and evidence of increased macular thickness on optical coherence tomography.

**Results:**

The overall rate of cystoid macular edema was 0.82%. Treatment by postoperative generic ketorolac 0.45% and prednisolone 1% demonstrated the highest rate of cystoid macular edema development (2.20% of the cases). Postoperative name-brand ketorolac 0.45% and prednisolone 1% exhibited intermediate rates of cystoid macular edema development (0.90% of the cases). Postoperative administration of bromfenac 0.09% and prednisolone 1% exhibited intermediate rates of cystoid macular edema development (0.44% of the cases). Preoperative and postoperative bromfenac 0.09% alone resulted in the lowest rate of cystoid macular edema development (0.09% of the cases). The rate of cystoid macular edema was significantly lower when bromfenac was used alone vs. either regimen where ketorolac and prednisolone were used (OR 0.043, 95% CI 0.002 to 0.312; p<0.001).

**Conclusions:**

Post-cataract surgery cystoid macular edema developed less frequently following topical non-steroidal anti-inflammatory drugs regimen compared to the other therapies evaluated. Bromfenac, without corticosteroids, achieved lower rates of cystoid macular edema vs. various combinations of nonste roidal anti-inflammatory drugs with corticosteroids.

## Abstract (pt)

**Objetivo:**

Avaliar a taxa de desenvolvimento do edema macular cistóide em pacientes submetidos à cirurgia de catarata em quatro esquemas terapêuticos diferentes.

**Métodos:**

O presente estudo é uma análise retrospectiva de 5.380 olhos após facoemulsificação não complicada na Wake Forest University. O período do estudo foi entre julho de 2007 e dezembro de 2012. Os pacientes receberam um dos quatro esquemas: cetorolaco genérico pós-operatório 0,4% e prednisolona 1%, cetorolaco 0,45% pós-operatório e prednisolona 1%, bromfenac 0,09% e a prednisolona 1% pós-operatório, bromfenaco 0,09% no pré-operatório e isoladamente no pós-operatório. Uma análise estatística foi realizada para avaliar as diferenças na taxa de desenvolvimento do edema macular cistóide entre os quatro diferentes regimes terapêuticos. O diagnóstico de edema macular cistóide exigiu uma piora da visão e uma evidência de aumento da espessura macular na tomografia de coerência óptica.

**Resultados:**

A taxa global de edema macular cistóide foi de 0,82%. O tratamento com cetorolaco genérico pós-operatório 0,45% e prednisolona 1% demonstrou a maior taxa de desenvolvimento de edema macular cistóide (2,20% dos casos). O cetorolaco 0,45% e a prednisolona 1% no pós-operatório exibiram taxas intermediárias de desenvolvimento de edema macular cistóide (0,90% dos casos). A administração de bromofenac 0,09% e de prednisolona 1% no pós-operatório apresentou taxas intermediárias de desenvolvimento de edema macular cistóide (0,44% dos casos). O bromfenac 0,09% no pré e pós-operatório isoladamente resultou na menor taxa de desenvolvimento de edema macular cistóide (0,09% dos casos). A taxa de edema macular cistóide foi significativamente menor quando o bromfenac foi utilizado isoladamente em relação ao esquema onde cetorolaco e a prednisolona foram usados (OR 0,043, 95% CI 0,002 a 0,312; p<0,001).

**Conclusões:**

O edema macular cistóide pós-cirurgia de catarata desenvolveu-se com menor frequência após o tratamento tópico de medicamentos anti-inflamatórios não esteroidais, comparado às outras terapias avaliadas. Bromfenac, sem corticosteróides, alcançou taxas mais baixas de edema macular cistóide vs. Várias combinações em comparação com as várias combinações de drogas anti-inflamatórias não esteroidais com corticosteróides.

## INTRODUCTION

Cataract is the leading global cause of blindness. Cataract surgery is one of the most common operations performed worldwide^(1)^. A serious side effect of cataract surgery is a surgical inflammatory response, such as cystoid macular edema (CME)^(2)^. The risk of CME’s development appears to be lower with phacoemulsification cataract surgery than with either extracapsular cataract extraction or intracapsular cataract extraction^(2)^. The outcomes of cataract surgery have been significantly improved by recent advances in surgical techniques. However, CME remains one of the most prevalent causes of postoperative visual decline following an uneventful cataract surgery^(3)^. To date the definitive mechanism involved in CME’s development has not been identified. A current leading theory of pathogenesis involves surgical trauma to intraocular tissues, which induces the release of prostaglandins and other inflammatory mediators^(3)^. An elevated concentration of inflammatory mediators increases the permeability of perifoveal capillaries and disrupts the blood-retinal barrier^(4)^. Subsequently, the pathologic hyperpermeability of retinal blood vessels and compromised blood-retinal barrier allow fluid leakage across the retinal vessel wall. This in turn causes the cystic accumulation of extracellular intra-retinal fluid in both the retina’s outer plexiform and inner nuclear layers^(5)^. CME may develop four to six weeks into the postoperative period. It is responsible for temporary or permanent vision loss^(6)^.

Given the knowledge that intraocular inflammation plays a role in the development of CME, a mainstay of treatment post-cataract surgery is the reduction of inflammation. To this end, several therapeutic regimens of topical anti-inflammatory medications are available. Two of the currently available drug groups used to control intraocular inflammation are corticosteroids and non-steroidal anti-inflammatory drugs (NSAIDs). In a meta-analysis, Rossetti et al. found a positive therapeutic effect of NSAIDs and corticosteroids in the prevention and treatment of CME^(7)^. It has been shown that NSAIDs are as powerful as corticosteroids to diminish postoperative inflammation, with an additional benefit if combination with standard corticosteroid postsurgical therapy^(8)^. In order to ensure a favorable outcome in patients undergoing cataract surgery, choice of the anti-inflammatory agent to be used is important. The purpose of the present study was to evaluate the rate of CME development among cataract surgery patients on four different therapeutic regimens: postoperative administration of generic ketorolac 0.4% and prednisolone 1%, postoperative administration of name-brand ketorolac 0.45% (Acular) and prednisolone 1%, postoperative administration of bromfenac 0.09% and prednisolone 1%, and preoperative and postoperative administration of bromfenac 0.09% alone.

## METHODS

The present retrospective chart review study was approved by the Wake Forest Baptist Health institutional review board. The study was performed in accordance with the tenets of the Declaration of Helsinki. We enrolled in the study 5,393 eyes of patients who underwent routine cataract surgery with intraocular lens placement. Surgeries were performed by three surgeons in the John Galt Outpatient Surgery Center at the Wake Forest Baptist Medical Center Department of Ophthalmology (Winston-Salem, North Carolina, USA). The study period went from July 2007 to December 2012. All cataract surgeries were performed using phacoemulsification under topical local anesthesia. All surgeons had equivalent years of experience and performed surgery using similar equipment, techniques, and small intraocular lenses in subjects from the same patient population. CME was diagnosed based on vision worsening and evidence of macular thickness’ increase on optical coherence tomography (OCT) post-cataract surgery. Typically, an OCT was ordered exclusively if the patient’s vision declined post-cataract surgery. Additional data collected included the following: patient demographics, date of cataract surgery, date of CME diagnosis, OCT confirmation of CME, and preoperative, perioperative, and postoperative therapeutic regimens.

The therapeutic regimens varied among the three surgeons. If the patient had a history of CME or was at high risk of developing CME, surgeon #1 prescribed a 5-week taper of postoperative name-brand prednisolone 1% (Pred Forte, Allergan, Irvine CA) accompanied by name-brand ketorolac 0.45% (Acular, Allergan, Irvine CA). No generic substitutions were allowed. If patient had a history of CME or was at high risk of developing CME, surgeon #2 prescribed a 5-week taper of postoperative generic prednisolone 1% and generic ketorolac 0.45%. Surgeon #3 prescribed two different therapeutic regimens during the five-year period. If the patient had a history of CME or was at high risk of developing CME, before September 2010, surgeon #3 prescribed a 5-week taper of postoperative name-brand prednisolone 1% and bromfenac 0.09%. After September 2010, surgeon #3 prescribed once daily bromfenac 0.09%, two days preoperative and continued for one month postoperatively. Surgeon #3 did not prescribe corticosteroids after September 2010 regardless of the risk for CME, difficulty of surgery, diabetes or use of iris manipulating devices.

Fisher’s Exact Test was used to assess the differences in CME’s development rate among the four different therapeutic regimens. P-values<0.01 were considered as statistically significant. Additionally, Fisher’s Exact Test was used to pool the three regimens involving steroids and the selective use of NSAID and compared to the NSAID only group.

## RESULTS

In the present study a total of 5,393 uncomplicated consecutive cataract surgery cases were enrolled. Among them, 13 cases were excluded due to the following reasons: baseline retinal abnormalities (n=10), inadequate follow-up (n=1), retained lens fragment from a previous surgery (n=1), and concomitant trabeculectomy (n=1). Following exclusion, 5,380 cases were included in the statistical analyses. Of these, 45.3% of cases were performed by surgeon #1, 13.5% by surgeon #2, and 41.2% by surgeon #3. Table 1 provides the descriptive details and lists the number of cases subdivided among the four different therapeutic regimens.

**Table 1:** Description of the peri-operative drops regimens used

Regimen (# eyes)	Drops utilized, frequency, and timing
1 (2,437)	Post-op Pred Forte taper, 5 weeks, no generics allowed Add Ketorlac QID post op if history of CME
2 (725)	Post-op prednisolone taper Ketorolac for patients with history of CME Generic substitutions allowed for steroid and NSAID
3 (1,128)	Post op branded prednisolone taper, 35 days Bromfenac on high risk patients (BID, 1 month) No generic substitutions
4 (1,090)	Bromfenac on every patient: QD 2 days pre-op until 1 month post-op No steroid No generic substitutions

**Figure 1:**
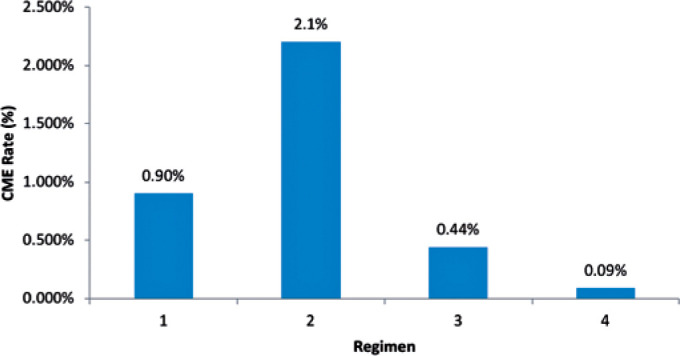
The rates of CME are depicted for each drop regimen. Regimen 1: 0.90% (23/2437). Regimen 2: 2.1% (15/725). Regimen 3: 0.44% (5/1128). Regimen 4: 0.09% (1/1090).

Of the 5,380 cases, 44 suffered from a postoperative visual decline and evidence of retinal thickening on OCT. Such developments occurred during the 1-3 months postoperatively. This yielded an 0.82% overall rate of CME for the study population. Figure 1 displays the rate of CME for the four different therapeutic regimens. Of note, therapeutic regimen #2, had the highest rate of CME (5/725 surgeries, 2.1% incidence), while therapeutic regimen #4, displayed the lowest rate of CME (1/1090 surgeries, 0.09% incidence). Therapeutic regimens #1 and #3, had intermediate rates of CME of 0.90% (23/2437 surgeries, 0.90% incidence and 5/1128, 0.44% incidence, respectively). Table 2 summarizes the demographics of the individuals included who developed CME for each therapeutic regimen.

**Table 2:** Cystoid macular edema with each regimen.

Demographics						
**Drop regimen**	**Number of** **cases**	**Average _ age**	**Range**		**Sex**	
			**Min**	**Max**	**M**	**F**
**1**	**23**	**72.1**	**33**	**91**	**11**	**12**
**2**	**15**	**75.2**	**60**	**86**	**2**	**13**
**3**	**5**	**74.2**	**68**	**87**	**1**	**4**
**4**	**1**	**87.0**	**87**	**87**	**0**	**1**
**Total**	**44**	**73.5**	**33**	**91**	**14**	**30**

Table 3 provides a comparison for rate of CME development among the four different therapeutic regimens, as well as the comparative statistics. Of note, Fisher’s exact test showed that the rate of CME development was significantly lower in therapeutic regimen #4 vs. therapeutic regimen #1 and #2. More importantly, when pooling the three corticosteroid regimens together, CME developed in 43/4290 cataract surgeries (1.0%) compared to 1/1090 cases in the NSAID alone group (0.09%). As described in table 4, findings show a statistically significant approximate 11-fold reduction in CME (p=0.001).

**Table 3:** Statistical comparison of various regimens.

Comparison	CME outcome, No. (%)	Fisher’s Exact Test (p-value)	Odds ratio, 95% confidence interval
Regimen 1 v 2	23 (0.90%) v 15 (2.21%)	0.011	0.422, (0.213-.842)
Regimen 1 v 3	23 (0.90%) v 5 (0.44%)	0.64	ns
Regimen 1 v 4	23 (0.90%) v 1 (0.09%)	0.005	0.096, (0.005-0.668)
Regimen 2 v 3	15 (2.21%) v 5 (0.44%)	0.002	4.7, (1.6-15.0)
Regimen 2 v 4	15 (2.21%) v 1 (0.09%)	<0.001	0.043, (0.002-0.312)
Regimen 3 v 4	5 (0.44%) v 1 (0.09%)	0.22	ns

**Table 4:** Steroid with selective NSAID (pooled regimens 1-3) vs. NSAID alone (regimen 4)

	Steroid ± NSAID	NSAID only	Fisher’s Exact Test
No post op CME	4247	1089	p=0.001^*^
CME cases, No. (%)	43 (1.02%)	1 (0.09%)	
Totals	4290	1090	

* = odds ratio for CME in the steroid ± NSAID group is 11.0 (95% CI of 1.5, 80.2).

## DISCUSSION

The present large observational study compared the effects of various therapeutic regimens on CME’s occurrence prevention following uncomplicated cataract surgery by phacoemulsification. Our results demonstrate significant differences in rate of CME development among cataract surgery patients on a wide variety of therapeutic agents in combination or alone. The present investigation’s novelty lies in the demonstration of variance in rate of CME development among cataract surgery patients on different therapeutic regimens. Based on this study’s findings, it appears that the choice of medical prophylaxis has a direct effect on CME’s occurrence prevention. To the best of our knowledge, this is the first study demonstrating that a single therapy regiment of a preoperative NSAID prolonged 1 month postoperatively culminated in the lowest CME rate, vs. the routine use of prednisolone with the selective addition of NSAIDs for certain cases. Additionally, of note, as to the different options of NSAIDs available, our analyses identified therapeutic regimens that allowed for generic substitutions to have a higher rate of CME development at 2.1% (regimen 2). Based on the evidence presented here, the prophylactic preoperative use of bromfenac beginning 2 days pre-cataract surgery and extending 1 month after the postoperative period exhibited the most optimal reduction in the incidence of CME at 0.09%, even in absence of corticosteroids.

In line with previously published reports, our analyses found the incidence of postoperative CME to be 0.82%. Specifically, earlier studies have estimated the rate of clinical CME to vary between 0.1 and 2%^(8^-^14)^. CME’s incidence changes among studies depending on how it is defined. While fluorescein angiography has been the traditional gold standard for CME’s detection, OCT may be more clinically relevant^(15)^. Specifically, OCT is a high-resolution, cross-sectional imaging modality that directly and reproducibly measures macular thickness^(16)^. Our methodology utilized OCT over angiographic detection of CME due to the several advantages it offers. The angiographic grading of CME is subjective, qualitative, and invasive. Presence of leakage on fluorescein angiography correlates poorly with visual acuity^(15)^. On the contrary, the OCT grading of CME is noninvasive and allows the objective quantification of CME’s spectrum by measuring changes in the retina’s volume^(11)^. Additionally, retinal thickening corresponds better with visual acuity^(17)^. Importantly, a previous study demonstrated that spectral-domain OCT provided higher sensitivity, specificity, and reproducibility for detection of CME, compared to fluorescein angiography. Specifically, the authors showed that spectral-domain OCT had a 96% sensitivity and a 100% specificity for CME’s detection^(11)^.

Interestingly, our study showed that a single anti-inflammatory agent of topical bromfenac had the lowest risk of CME, even in the absence of corticosteroids, compared to the selective postoperative administration of NSAIDs (including bromfenac, combined with corticosteroids). These findings suggest that a consistent use of a therapeutic scheme involving bromfenac, has a positive effect on CME’s prevention. To this end, therapy should be initiated 2 days prior to cataract surgery. Seemingly contradictory, a previous meta-analysis that examined the association between NSAIDs’ administration and CME’s incidence found no long-term benefits of NSAID therapy in CME’s prevention after cataract surgery^(4)^. We believe that the factors leading to this discrepancy are two. First, this meta-analysis included only a single article in which bromfenac was used, and importantly that study used it only postoperatively. Second, there was a lack of power caused by insufficient cases involving modern small-incision phacoemulsification. Specifically, only 60 eyes were included in each group. Our study differs from the other studies examined in the metaana lysis. Specifically, we included over 1000 eyes with small-incision phacoemulsification, in which bromfenac was the sole anti-inflammatory agent. Furthermore, it has been established that there exists a difference in anti-inflammatory activity between all of the NSAIDs. Bromfenac’s advantage lies in that it contains a bromine atom that provides an approximate tenfold greater lipophilicity. This in turn improves penetration into ocular tissue, allowing for lengthier effects^(18)^. Specific NSAIDs, like bromfenac, have better penetration into ocular tissues vs. lipophobic corticosteroids. Additionally, to be effective, they only require penetration into the cytoplasm and not the nucleus.

Different classes of anti-inflammatory medications block varying portions of the inflammatory cascade, triggered by trauma to the ocular tissue during cataract surgery. Corticosteroids inhibit both the cyclooxygenase and lipoxygenase pathways by reduction of DNA synthesis. As a consequence, they reduce the downstream production of prostaglandins and leukotrienes^(19)^. In contrast, NSAIDs directly inhibit the cyclooxygenase pathway, blocking COX-1 and COX-2 enzymes. Therefore, they suppress prostaglandins’ synthesis^(3)^. In theory, based on their mechanism of action, corticosteroids seemingly possess broader anti-inflammatory properties than NSAIDs. However, a previous study directly comparing corticosteroids and NSAIDs has inconsistently observed differences in reduction of intraocular inflammation post-cataract surgery^(20)^. Such finding may be explained by the need to increase the frequency of topical steroids and/or by the lack of suppression, by corticosteroids, of any arachidonic acid present at surgery. In the present study a direct comparison of corticosteroids vs. NSAIDs was not performed. As a consequence assessment of the two anti-inflammatory medications’ effectiveness of in reducing intraocular inflammation cannot be performed. However, based on our experience, we believe that the consistent use of bromfenac alone appears to have better control in preventing CME in routine modern cataract surgery compared to consistent use of corticosteroids combined with selective use of an NSAID. Of note, in favor of NSAIDs, a previous study found NSAIDs to be more effective than topical corticosteroids at re-establishing the blood-aqueous barrier^(3)^. Importantly, an additional confounding factor may be linked to generic medications. Specifically, the liberal use of generics resulted in higher rates of postoperative CME in our study. This finding suggests that generic ketorolac and prednisolone may have a lower degree of reduction of intraocular inflammation post-cataract surgery. Such consequence may be due to toxicity and/or compliance issues^(21)^.

NSAID’s treatment timing may also influence the risk of CME’s development post-cataract surgery. However, to date consensus guidelines for the timing of proper perioperative topical NSAIDs have not been established^(6)^. Our results suggest that prophylactic preoperative bromfenac continued one month postsur gery performed greater than selective postoperative NSAIDs combined with routine corticosteroid. Based on this evidence, it is possible to speculate that topical NSAIDs, if initiated preoperatively and continued postoperatively, may alone substitute for postoperative corticosteroids in the treatment of post-cataract surgery inflammation. It is known that prostaglandins have short half-lives. However, clinically important inhibition of cyclooxygenase enzymes probably requires sustained inhibitory drug levels. These are not obtained immediately after the initial application^(4)^. Therefore, continual use of NSAID starting several days pre-surgery may be needed in order to achieve sustained intraocular levels sufficient to inhibit meaningful prostaglandin synthesis at the time of surgery^(4)^. Alternatively, it is possible that the superior penetration of bromfenac combined with pretreatment successfully controls both postoperative inflammation and CME.

NSAIDs provide numerous distinct clinical advantages over corticosteroids. Specifically, it has been shown that they are responsible for the stabilization of intraocular pressure, maintenance of intraoperative mydriasis, minimization of endothelial cell loss, provision of postoperative analgesia, and reduction of the risk of secondary infections^(2^,^4^,^8)^. Such beneficial effects of NSAIDs are in addition to their ability to control postoperative inflammation and prevent CME occurrence. Conversely, corticosteroids have been associated with a number of side effects. Of note, it has been shown that they are responsible for elevations of intraocular pressure, corneal thinning, slow wound healing, and an increased risk of ocular infection due to inhibition of the normal immune response^(13)^. Based on these drawbacks of corticosteroids, and on our evidence suggesting an 11 fold reduction in CME with bromfenac monotherapy, we believe that bromfenac is a safer alternative for the treatment of inflammation following modern phacoemulsification cataract surgery. Such observation holds true despite our long-standing tradition of including a corticosteroid with every cataract surgery.

Several limitations may be identified in the present study. First, a convenience sampling from a universityba sed general ophthalmology clinic was utilized. Specifically, the study subjects were not population-based. As a consequence they should not be expected to represent the general population. Therefore, this study’s findings should not be generalized to patients possessing characteristics differing from those enrolled in the studies reviewed. Second, evaluation of the optimal timing for prophylactic treatment in the preoperative period was not performed. In the present study all preoperative NSAIDs were administered only 2 days pre-cataract surgery. Third, despite the three surgeons having used similar equipment and techniques, differences in clinical judgment and variable use of NSAIDs may be identified. Fourth, given the retrospective nature of the present study, it was not possible to control for certain preoperative factors contributing to the development of postoperative CME. In an earlier report, an electronic medical records review of 81984 eyes undergoing cataract surgery was performed on patients with diabetes. The study showed that diabetic patients have increased relative risk of postoperative CME^(9)^. Our study did not control for the effect of diabetes. However, we can assume that the numbers of diabetic patients may potentially be evenly distributed among the different study groups. Therefore, we would expect diabetes to exert similar effects in all groups. Our 3 surgeons did not employ different preoperative assessment criteria for patients’ selection for cataract surgery. In North Carolina, the prevalence of diabetes in adults is approximately 10%, which may be presentative of our study population^(22)^. A future prospective study may help clarify our findings by accounting for risk factors associated with increased CME incidence post-cataract surgery.

In summary, this study demonstrates that the preferred therapy to prevent CME post-cataract surgery involves the consistent use of an NSAID, presumably bromfenac. NSAID treatment preferably should commence a few days pre-surgery. Additionally, the use of corticosteroids is not necessarily needed. Preoperative and postoperative topical bromfenac significantly reduced the odds of developing postoperative CME. Specifically, the odds were reduced by 11-fold, as compared to combined selective postoperative NSAID, including bromfenac, with routine corticosteroids. Currently, topical therapy with both NSAIDs and corticosteroids are the mainstay in the prevention and treatment of postoperative CME. However, to date there are no clear guidelines for prophylaxis regimen. Every ophthalmologist has his or her own therapeutic strategy, with the use of various anti-inflammatory agents. Our findings suggest that, in order to help reduce CME’s incidence, a consistent use of preoperative NSAIDs should constantly be included in the prophylaxis regimen. Of note, in order to prevent CME, postoperative NSAIDs may be used alone or concurrently with corticosteroids. However, it is important to emphasize that our data suggests that treatment with exclusively bromfenac preand postoperatively may be effective in reducing CME’s incidence to almost zero levels. While our results suggest this finding, further prospective analysis is needed to verify our hypothesis. In conclusion, we believe that corticosteroids may not be absolutely essential after an uneventful cataract surgery. Especially given that corticosteroids are associated with several negative side effects (e.g. delayed wound healing, immunosuppression and raised intraocular pressure). In addition, bromfenac monotherapy is the most convenient therapy for the patient. Specifically, it is delivered as only one drop daily, no tapering is required, and it has less co-pays at the pharmacy.
